# Comparison of WHO laboratory-based and non-laboratory-based CVD risk charts among hypertensive adults attending primary healthcare centers in West Africa sub-region

**DOI:** 10.1371/journal.pone.0317640

**Published:** 2025-06-09

**Authors:** Kojo Awotwi Hutton-Mensah, Olayinka Rasheed Ibrahim, Adaku Nwankwo, George Bediako Nketiah, Funmi Temidayo Adeniyi, Abukari Yakubu Natogmah, James Ayodele Ogunmodede, Dike Ojji, Olumide Adesola, Biodun Sulyman Alabi, Olugbenga Ayodeji Mokuolu, Daniel Sarpong

**Affiliations:** 1 Directorate of Medicine, Komfo Anokye Teaching Hospital, Kumasi, Ghana; 2 Department of Pediatrics, Division of Clinical Medicine, University of Global Health Equity, Kigali, Rwanda; 3 Department of Internal Medicine, Gwarimpa General Hospital, Abuja, Nigeria; 4 Department of Family Medicine/Polyclinic, Korle Bu Teaching Hospital, Accra, Ghana; 5 Department of Pediatrics, University College Hospital, Ibadan, Nigeria; 6 St Anthony Ann Hospital, Donyina, Ghana; 7 Department of Internal Medicine, University of Ilorin, Ilorin, Nigeria; 8 Department of Internal Medicine, University of Abuja Teaching Hospital, FCT, Nigeria; 9 Institute of Child Health, University of Ibadan, Ibadan, Nigeria; 10 Department of ENT, University of Ilorin, Ilorin, Nigeria; 11 Department of Pediatrics, University of Ilorin, Ilorin, Nigeria; 12 Office of Health Equity Research, Yale School of Medicine, New Haven, Connecticut, United States of America; University of the Witwatersrand Johannesburg, SOUTH AFRICA

## Abstract

**Background:**

The World Health Organization (WHO) non-laboratory cardiovascular disease (CVD) risk chart is sub-region-specific and is advocated in resource-constrained settings. However, the extent of agreement with laboratory-based assessment among hypertensive adults attending primary health centers (PHCs) in the West Africa sub-region remains unknown. This study compared 10-year CVD risk among adults with hypertension attending PHCs in Ghana and Nigeria.

**Materials and methods:**

This cross-sectional study recruited 319 adults with hypertension at PHCs in Ghana and Nigeria. All participants had their blood pressure, anthropometrics, fasting blood sugar, and fasting cholesterol measured following standard procedures. WHO laboratory and non-laboratory CVD risks were assessed and compared using Kappa statistics, correlation, and Bland-Altman Plot,

**Results:**

The median (interquartile range) for laboratory-based and non-laboratory-based CVD risk scores were comparable [7.0 (4.0 11.0) vs. 7.0 (4.0 to 11.0), p = 0.914]. Of the 319 participants, laboratory-based assessment classified 214 (67.1%) as low risk, while 210 (65.8%) were classified as low risk using the non-laboratory method. Eleven (3.4%) and 14 (4.4%) participants were classified as high-risk using laboratory- and non-laboratory-based methods, respectively. Overall, there was a very good positive correlation between the CVD risk assessment methods (r = 0.948, p<0.001). For all participants combined, there was substantial agreement (Kappa statistics), with K = 0.766. Bland-Altman showed a mean bias of 0.15 (SD = 1.74) in favor of non-laboratory-based assessment of CVD with an upper limit of 3.57 and a lower limit of –3.26.

**Conclusion:**

There was substantial agreement between laboratory- and non-laboratory-based WHO CVD risk charts in this study. In low-resource settings, such as Ghana and Nigeria, the WHO non-laboratory CVD risk prediction model offers a huge opportunity for primary CVD prevention in adults with hypertension.

## Introduction

Cardiovascular disease (CVD) is the leading cause of death worldwide, accounting for 33% of all deaths with low- and middle-income countries like Ghana and Nigeria, accounting for as many as 80% of these CVD-related deaths [1]. In addition, the majority of CVD-related burden in Africa occurs in people between the ages of 30–70 years, and the resulting disability-adjusted life years (DALYs) poses serious economic and social consequences for families and the nation at large [2,3]. Consistent with global statistics, risk factors for CVD in Africa include hypertension, diabetes mellitus (DM), dyslipidemia, central obesity, tobacco use, physical inactivity, unhealthy diet, and hyperuricemia, most of which are preventable [4].

Considering the huge burden of CVD, as well as health inequity in Africa, emphasis must be placed on CVD prevention. Early detection is critical in disease prevention; hence, using CVD risk prediction tools may play significant role in CVD prevention.

There are many CVD risk prediction models and scores that have been developed, including the WHO CVD risk score, Framingham risk score, pooled cohort equation and systematic coronary risk evaluation, among others [5]. Currently studies on CVD risk estimation in West Africa have employed the use of CVD risk prediction models including WHO CVD risk score, Framingham risk score, pooled cohort equation, Globorisk in predicting CVD risk [6,7]. However, the sub-region-specific WHO CVD risk score chart has been advocated for use in West Africa to predict the 10-year risk of fatal and nonfatal CVD outcomes. It consists of non-laboratory and laboratory-based charts that are based on scores derived from parameters obtained from laboratory investigations (cholesterol and blood sugar), anthropometrics (body mass index), age, sex, and smoking status [8]. The laboratory-based chart utilizes information on age, sex, smoking status, systolic blood pressure (SBP), history or evidence of DM, and total cholesterol. In contrast, the non-laboratory-based chart includes information on age, sex, smoking status, SBP, and body mass index (BMI) without a requirement for any laboratory test. The aggregate scores (in percentage) obtained using either laboratory-based or non-laboratory-based chart are used to predict the likelihood of a patient’s risk of developing adverse CVD events within ten years and WHO advocates for early interventions in those with moderate to high risk [>10–20%] [8].

Due to resource limitations in low- and middle-income countries, WHO non-laboratory CVD charts are favored as they do not require laboratory tests, especially at primary healthcare levels where access to laboratory tests is limited [9]. Although the data for the derivation of the WHO-CVD risk charts were from various sub-region data, including the West Africa sub-region, the assessment of the model performance has not been advocated for various sub-populations due some inconsistency reported in earlier studies [10]. For instance, in the East Africa sub-region, a multi-country study that involved six nations showed that non-laboratory methods underestimated CVD risk by as much as 60% despite very good concordance (91.3%) when compared with laboratory-based methods in the diabetic sub-population [11]. Similarly, in Iran, at a high-risk group (score > 20%), the sensitivity of the WHO non-laboratory-based method was as low as 25% for males less than 60 years, showing its poor sensitivity as a screening tool [9]. In India, despite modest concordance [78%] between the two methods of assessment, the non-laboratory-based charts underestimated the CVD risk by 8.7% among the participants aged 40–74 years [12]. At present, with an estimated population of 460 million and a hypertensive prevalence of 27%, the extent of agreement between WHO laboratory and non-laboratory risk among West Africans with hypertension remains unknown [13,14]. Therefore, in this study, we compared the WHO non-laboratory-based CVD risk score with the WHO laboratory-based risk score among patients with hypertension attending PHCs in Ghana and Nigeria.

## Materials and methods

### Study design and settings

This cross-sectional descriptive study included adults with hypertension attending PHCs in Ghana and Nigeria. This study was conducted at the Okelele Primary Healthcare Center, Ilorin, Nigeria, and St. Anthony Ann Hospital, Donyina, Ashanti Region, Ghana, with both facilities located within urban regions. The two primary healthcare centers were comparable in levels of healthcare services offered, including provision of outpatient treatment and follow-up for adults with hypertension.

### Study participants

This study included patients aged 40–74 years with hypertension who consented to participate. Hypertension was defined as those already on treatment (controlled or uncontrolled), treatment-naïve with a blood pressure ≥ 140/90 mmHg, and newly diagnosed with three resting blood pressure measurements (BP ≥ 140/90 mmHg). We excluded adults with evidence of CVD (stroke, heart failure, peripheral artery disease, or ischemic heart disease, as the CVD risk assessment charts are designed to predict 10-year risk of adverse CVD events), pregnant women, and those that declined or did not complete the study.

### Sampling technique

We recruited patients from each of the sites using a systematic sampling technique. We invited every third registered hypertensive adult at each health facility to participate. If a patient declined consent or failed to meet the inclusion criteria, we invited the next patient on the list. We continued this process until we achieved the minimum sample size at each site. Additionally, we replaced participants who withdrew or did not complete the study (such as not submitting blood samples) with the next ones on the register.

### Sample size estimation

Using a prevalence of hypertension of 27% in the West Africa sub-region [13], at a 5% level of precision and a 95% confidence interval, we estimated a minimum sample size of 300 [15].

### Participant recruitment and Data collection

A total of 319 participants were consecutively recruited between 1^st^ July 2023 and 28^th^ December 2023, with 160 participants from Nigeria and 159 from Ghana. We used a pretested questionnaire adapted from WHO’s STEP-wise approach to non-communicable disease risk factor surveillance tool to gather participants’ relevant sociodemographic and cardiovascular risk factors. The STEPS survey questionnaire for non-communicable diseases is a standardized tool validated across various sub-regions, including West Africa. Each participant also underwent physical measurements, including blood pressure (BP) measurements.

### Physical measurements

Anthropometric measurements were performed according to the WHO guidelines. The weight was measured using an Omron HN286 electronic human weighing scale with an accuracy of 0.1 kg. Each participant’s height was measured with a “Seca 213” mobile stadiometer with an accuracy of 0.1 cm. BMI was calculated using the following formula: BMI (kg/m^2^) = weight (kg)/ height (m^2^).

We measured each participant’s BP using a validated upper-arm BP monitor (Omron M7 Intelli IT’). In brief, each participant sat quietly with their feet on the floor and their clothes loosened around the arm for at least five minutes before blood pressure readings were taken. The displayed BP readings were documented in the study protocol. We obtained three serial BP measurements three minutes apart, and used the average of the last two readings as a measure of BP values the data analysis.

### Cardiovascular disease (CVD) risk score assessment

We calculated the CVD risk scores of participants using WHO CVD (laboratory-based) prediction charts and non-laboratory-based charts (2019 revised edition) [8]. For laboratory-based prediction scores, each participant’s score was calculated based on age, sex, smoking status, presence or absence of diabetes, SBP, and total cholesterol. In the non-laboratory group, each participant had CVD risk scores based on age, sex, smoking status, SBP, and BMI.

### Outcomes

Comparison of laboratory-based CVD risk scores and non-laboratory-based risk scores in adults with hypertension attending PHCs in Ghana and Nigeria

### Data analysis

We analyzed the data from the study protocol using IBM SPSS version 29. Descriptive statistics were used to summarize participants’ sociodemographic variables. The WHO CVD risk scores did not follow a normal distribution and were presented as medians with interquartile ranges (IQR) and further compared using the Mann-Whitney U test. The continuous variables that were normally distributed (systolic BP, diastolic BP, cholesterol, and BMI) were compared using the independent T-test. Pearson’s correlation coefficients were used to assess the relationship between the two CVD risk score assessment methods. To assess the concordance and agreement between the two methods, we used Kappa statistics [which assessed the concordance between the risk stratifications as categorical variables assessed by the two CVD risk assessment methods] and Bland-Alman plots [which assessed agreement between two methods as continuous data], respectively. These two statistics tools were selected because neither of the CVD risk assessment measurements are gold standards. The Kappa statistics were interpreted as follows: < 0 indicated (less than chance agreement); 0.01 to 0.20 (slight agreement); 0.21 to 0.40 (fair agreement); 0.41 to 0.60 (moderate agreement); 0.61 to 0.80 (substantial agreement); and 0.81 to 0.99 (almost perfect agreement). The WHO CVD risk, which classifies the 10-year risk of CVD events, included risk scores < 5%, 5% to < 10%, 10% to < 20%, 20% to < 30%, and ≥ 30%, which indicated very low-, low-, moderate, high-, and very high-risk groups, respectively. The level of statistical significance was set at p < 0.05.

### Ethical approval

This study was approved by the Ghana Health Service Ethics Review Committee (GHS-ERC:007/05/23) and the Kwara State Ethical Review Committee (ERC/MOH/2023/02/090). We also sought permission from the appropriate authorities at both PHC facilities. A detailed explanation of what the study entailed in information sheets, including study procedures, was made available to all participants in the language they best understood, and written informed consent was obtained. The data collected were coded to ensure the anonymity of the study participants and stored in a password-encrypted computer.

## Results

### General characteristics

A total of 319 adults with hypertension receiving care at PHCs in the two countries (160 from Nigeria and 159 from Ghana) participated in this study. Participants’ mean (standard deviation) age was 59.1 (10.2) years. This study included 259 women (81.2%). Twenty-three (7.2%) participants had diabetes mellitus, and five smoked cigarette (Table 1). The median (interquartile range-IQR) for laboratory-based CVD risk scores was 7.0 (4.0, 11.0) and was comparable to the non-laboratory-based CVD risk scores [7.0 (4.0, 11.0)] (p = 0.914) (Table 1).

**Table 1:** General characteristics of the study participants.

Variables	Total	Males (n = 60)	Females (n = 259)	P values
Age group [Years]				
≤ 50	74 (23.2)	15	59	0.931*
51-60	94 (29.5)	17	77	
>60	151 (47.3)	28	123	
Smoke cigarette	5 (1.6)	3	2	0.048f**
Alcohol intake	19 (6.0)	12	7	<0.001f**
Diabetes Mellitus	23 (7.2)	5	18	0.781f**
Systolic BP	142.8 (24.4)	144.4 (24.3)	142.4 (24.4)	0.579***
Diastolic BP	86.4 (13.1)	86.2 (13.9)	86.4 (14.0)	0.914***
Cholesterol	5.2 (1.1)	4.7 (1.0)	5.3 (1.1)	<0.001***
BMI [Kg/m2]	26.5 (5.6)	25.2 (4.9)	26.8 (5.7)	0.039***
Laboratory-based CVD risk scores	7.0 (4.0 to 11.0)	7.0 (4.0 to 13.8)	7.0 (4.0 to 11.0)	0.937#
Non-lab. based CVD risk scores	7.0 (4.0 to 11.0)	7.0 (4.0 to 12.0)	7.0 (3.0 to 11.0)	0.923

BP-Blood pressure; BMI-Body mass index; CVD-cardiovascular disease, *Chi-square; **Fischer exact test,*** T-test, # Mann-Whitney U test.

### CVD risk scores classification based on the laboratory and non-laboratory assessments

**Fig 1:**
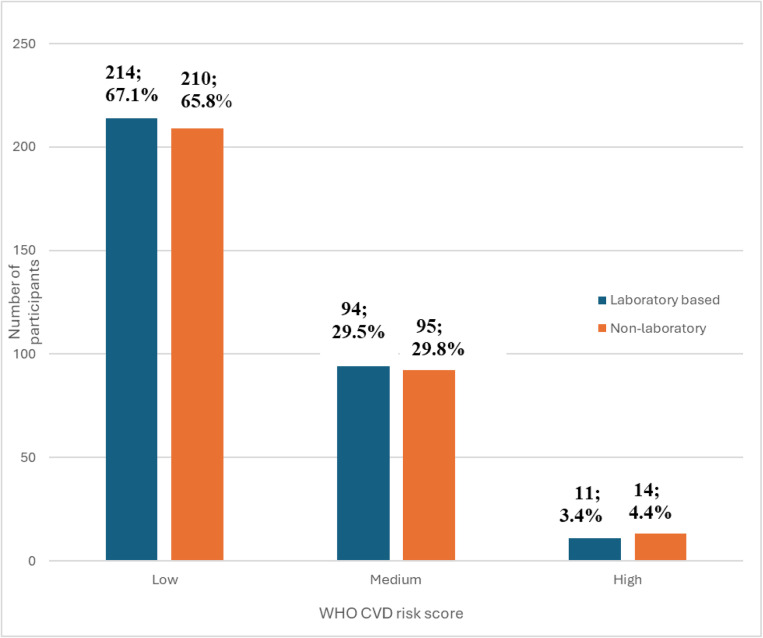
Classification of CVD risks among the study participants.

Of the 319 participants in this study, laboratory-based assessment classified 214 (67.1%) as low risk, while 210 (65.8%) were classified as low risk using the non-laboratory method. Eleven participants (3.4%) were classified as high-risk using laboratory-based assessment, whereas 14 (4.4%) were classified as high-risk using non-laboratory-based methods (Fig 1).

### Correlation of the CVD risk scores between laboratory and non-laboratory assessments in the study participants

Overall, there was a strong positive correlation between the two CVD risk assessment methods (r = 0.948, p < 0.001). Based on the age stratification, participants aged 50 years and below had a good positive correlation. In contrast, those older than 50 had a strong positive correlation between laboratory and non-laboratory-based assessments (Table 2).

**Table 2:** Correlation of the CVD risk scores between laboratory and non-laboratory assessments in the study participants.

Variables	Total	*r*	95% CI	p
**Age groups [Years]**				
**40-74 (all)**	319	0.948	0.936, 0.958	<0.001
**≤ 50**	74	0.716	0.583, 0.812	<0.001
**51-60**	94	0.868	0.807, 0.910	<0.001
**>60**	151	0.893	0.855, 0.921	<0.001
**Sex**				
**Male**	60	0.938	0.898, 0.963	<0.001
**Female**	259	0.952	0.939, 0.962	<0.001

### Agreement between the laboratory and non-laboratory-based risk scores

**Fig 2:**
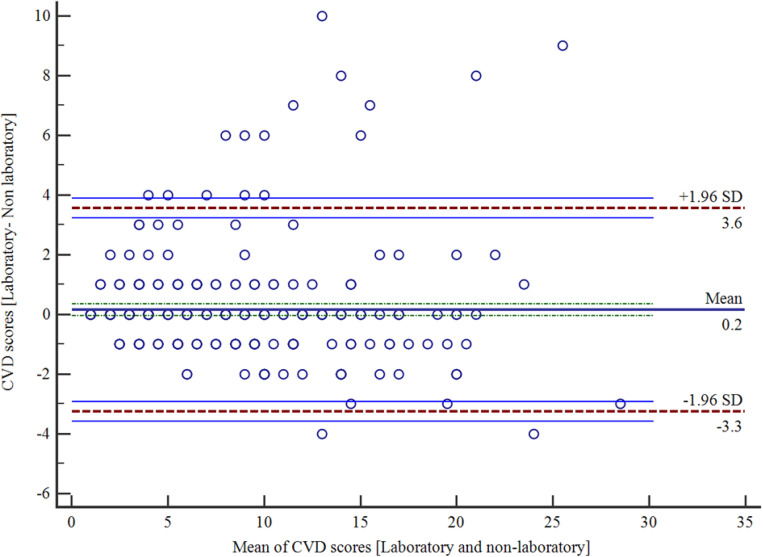
Bland-Altman plot showing agreement between the laboratory and non-laboratory assessment of CVD risk scores.

For all participants combined, there was substantial agreement (Kappa statistics), with K = 0.766. Similarly, in both males and females, the kappa statistics showed good agreement (Table 3). For those that were aged 60 and below, the Kappa statistics showed fair agreement [0.234, p = 0.002]. Bland-Altman shows a mean bias of 0.15 (SD = 1.74) in favor of non-laboratory-based assessment of CVD with an upper limit of 3.57 and a lower limit of −3.26 (Fig 2).

**Table 3:** Agreement between laboratory and non-laboratory-based risk scores.

Variables	Non-laboratory-based CVD risk				
Laboratory-based CVD risk (All)	low	moderate	High	Total	Kappa (SE)
**All**					
Low	198	16	0	21	0.766 (0.037), p < 0.001
Moderate	12	77	5	94	
High	0	2	9	11	
Total	210	95	14	319	
**Male**					
Low	35	2	0	37	0.775 (0.079), p < 0.001
Moderate	3	14	1	18	
High	0	1	4	5	
Total	38	17	5	60	
**Female**					
Low	163	14	0	177	0.763 (0.041), p < 0.001
Moderate	9	63	4	76	
High	0	1	5	6	
Total	172	78	9	256	
**≤ 60 years**					
Low	155	7	0	162	0.234 (0.154), p = 0.002
Moderate	4	2	0	6	
High	0	0	0	0	
Total	159	9	0	168	
**> 60 years**					
Low	43	9	0	52	0.708 (0.055), p < 0.001
Moderate	8	75	5	88	
High	0	2	9	11	
Total	51	86	14	151	

SE-Standard error.

## Discussion

The rising burden of CVD coupled with low health expenditure in sub-Saharan Africa remains a critical challenge that requires effective intervention [1]. One key strategy to effectively combat CVD is to reduce its incidence through robust and effective screening aimed at identifying those at risk of adverse outcomes using CVD risk prediction models at primary healthcare levels, which are closer to the community. In this study, we compared the non-laboratory-based WHO CVD risk chart with the laboratory-based WHO CVD risk chart in hypertensive adults attending PHCs in Nigeria and Ghana.

Most participants in this study were at ‘low risk’, with a median risk score of 7% for both laboratory- and non-laboratory-based. This finding is similar to other studies in Bhutan [16], India [12], Peru [17], and East Africa [11], where the average CVD risk scores ranged from very low-risk to low-risk among the studied cohort. A study by Wagner et al in 4 African countries showed that among the rural Ghanaian cohort, the CVD risk estimate of 2.6% and 2.9% using the laboratory and non-laboratory WHO CVD risk chart respectively was lower than the 7% obtained in our study [6]. This difference could be because in our study all participants had at least one risk factor for CVD compared to their study in which 17% of participants had no risk factor for CVD. Also, our study was conducted in an urban setting which has a higher prevalence of CVD risk factors compared to the rural setting in which the study by Wagner et al was conducted [18]. Also, the findings in our study are contrasted by findings of a study in Bangladesh in which the average CVD risk score obtained was 10.3%, suggesting a moderate CVD risk [19]. The differences between our study and that of Bangladesh may be due to variations in lifestyle, genetics, and socioeconomic factors in different populations [20].

Our present study also shows that the non-laboratory risk chart predicted a slightly higher number of participants at high risk compared with laboratory-based assessment (4.4% vs. 3.4%), which is similar to a study in Bhutan, where the non-laboratory-based chart predicted 0.39% of participants as high risk compared to 0.23% as per the laboratory-based chart [16]. This may be explained by the fact that both studies had a low prevalence of diabetes and smoking, which has been identified to impact risk stratification using non-laboratory methods significantly. This justification is further corroborated by a study in the East African sub-region, which showed that non-laboratory-based risk charts are suitable for people without diabetes in low-resource settings [11] Other possible reasons for our observed differences between the two methods of CVD risk assessment with a higher classification for high-risk by non-laboratory-based method may be due to recruitment of homogenous of adults with hypertension at the entry point, which may weight more in the models. Also, the overall mean cholesterol, a key component of laboratory model was normal in this study, which could be impacted on the laboratory assessment. Also, the observed differences between the two methods regarding high-risk group for CVD adverse events show that the methods of assessment should be clearly indicated while stratifying and following up patients. In addition, at a higher health facility, combination of both tools may allow to choose highest risk stratification for adults with hypertension.

Also, worthy of noting in this study is the implication of the better performance of non-laboratory-based methods compared with laboratory-based methods in this study, which shows that in West-Africa sub-region, the concern of under-performance of non-laboratory may not be present. Hence, the utilization of non-laboratory based can easily be encouraged across the primary healthcare facilities in the sub-region allowing for risk stratification and monitoring of adults with hypertension. In addition, the non-laboratory risk assessment tool does not require any laboratory tests, and we advocate for its implementation to stem the burden of CVD adverse outcomes in the sub-region.

Our study showed substantial agreement between the two risk charts for both sex and age, with kappa statistics of 0.76, corresponding to substantial agreement. According to global work conducted by the WHO, there was a moderate agreement between the WHO 2019 CVD risk score using laboratory and non-laboratory algorithms [8]. The findings of this study are comparable to findings from the ‘Fasa’ cohort study in Iran [9] as well as a cross-sectional study in Bhutan [16], where there was substantial agreement between the two risk scores with a kappa of 0.68 and 0.74, respectively, and among the North Indian population with a kappa statistic of 0.64 [12]. The substantial agreement obtained in this study is similar to the substantial agreement of 0.78 obtained among people with normoglycemia and impaired fasting glucose in East Africa [11]. However, the same study observed a moderate agreement of 0.46 in the Eastern African cohort with diabetes [11]. The differences with respect to the diabetes group in East Africa may be due to the low number of adults with diabetes in this study (n = 23), and hence the minimal impact on the overall agreement between the two methods. The substantial agreement between the laboratory-based WHO CVD risk and the non-laboratory-based risk assessment has great implications for primary prevention of CVD in low-resource settings, such as the West Africa sub-region with low health expenditure per capita. Thus, this study supports using a simple and inexpensive non-laboratory-based WHO CVD risk chart for CVD risk screening at PHCs in the West Africa sub-region. This will facilitate the identification of those at high risk among hypertensive adults and ensure prompt early referral to higher levels for intervention.

In this study, the Bland-Altman analysis showed a mean bias of 0.15 in favor of the non-laboratory risk chart and the 95% limits of agreement ranging from −3.26 to 3.57, which denotes a reasonable agreement. The positive mean bias close to zero implies that the two methods generally agree, with minimal systematic differences. With a 95% confidence interval, including 0 implies minimal systematic difference (0.15), suggesting the difference is not statistically significant, and the methods agree. Our findings compare favorably with those of a study in Bangladesh [19] and an East African cohort study [11]. While we did not perform a sub-analysis to explore the extent of agreement among sub-populations in this study, the limits of agreement obtained suggest that among hypertensive adults in Nigeria and Ghana, non-laboratory methods can be used in place of laboratory methods. Our study also showed a strong positive correlation (r = 0.948) between laboratory- and non-laboratory-based WHO CVD risk charts. The correlation was better in participants who were greater than 50 years old than in those who were less than 50 (0.880 vs. 0.716). This finding is corroborated by a population-based study using in Iran, where there was a robust correlation between laboratory-based and non-laboratory tests, with the correlation coefficient being higher in the elderly [21].The correlation between the laboratory and non-laboratory based WHO CVD risk charts may be higher in the increasing age (>45 years for men; > 65 years for women) because age is an important independent risk factor for CVD and weighs heavily in calculating CVD risk in both charts; hence as the age increases it independently contributes significantly to the calculated CVD risk scores using both charts [22,23]. A strong positive correlation between laboratory and non-laboratory WHO CVD risk charts underscore the accuracy of non-laboratory-based risk charts in predicting CVD risk. This provides an opportunity to address the unmet need for inexpensive and readily available risk prediction models for estimating the 10-year CVD risk in people seeking medical care at PHCs.

This is the first multi-country study in the West African sub-region to examine and compare laboratory-based and non-laboratory CVD risk charts at primary healthcare levels, where many of the populace receive care. Other strengths of this study include using multiple and appropriate statistical methods to compare the two risk charts, including the Kappa statistic, Bland–Altman plots, and correlation. Despite these strengths, this study had some limitations. The sample size might limit the generalization of the findings to Ghanaian and Nigerian populations. In addition, this study was cross-sectional; hence, causality could not be established between the observed CVD risk scores and future adverse CVD events, which could have allowed us to assess the real-time performance of each of the CVD risk assessments. We also did not analyze some potential confounders such as dietary pattern and socioeconomic status, that may influence CVD risk. Our sample size was not designed to detect significant differences in some sub-population such as people with diabetes mellitus, which were relatively small (n = 23) in this study and hence making our findings less generalizable to this sub-population.

## Conclusion

This study highlights that the cardiovascular disease (CVD) risk among hypertensive patients attending primary health centers (PHCs) in Ghana and Nigeria is relatively low, as indicated by the World Health Organization (WHO) CVD risk chart. Notably, there is strong agreement between laboratory-based and non-laboratory-based assessments. In resource-limited settings like Ghana and Nigeria, adopting a low-cost, non-laboratory CVD risk prediction model offers a vital opportunity for primary prevention of CVD. By utilizing these methods at the PHC level, healthcare providers can swiftly identify high-risk patients and refer them for timely interventions, significantly reducing the burden of CVD. For optimal health outcomes in West Africa, policymakers should implement non-laboratory-based WHO CVD risk charts. This will empower healthcare workers to efficiently screen hypertensive patients and refer those at high risk for further evaluation, fostering a proactive approach to cardiovascular health in the region.

## Supporting information

S1 File Who CVD Risk Assessment_Data_Base_plos. (XLSX)
